# Molecular Basis for the Sorting of the SNARE VAMP7 into Endocytic Clathrin-Coated Vesicles by the ArfGAP Hrb

**DOI:** 10.1016/j.cell.2008.07.023

**Published:** 2008-09-05

**Authors:** Paul R. Pryor, Lauren Jackson, Sally R. Gray, Melissa A. Edeling, Amanda Thompson, Christopher M. Sanderson, Philip R. Evans, David J. Owen, J. Paul Luzio

**Affiliations:** 1 Cambridge Institute for Medical Research and Department of Clinical Biochemistry, University of Cambridge, Addenbrooke's Hospital, Hills Road, Cambridge CB2 0XY, UK; 2 Medical Research Council Laboratory of Molecular Biology, Hills Road, Cambridge CB2 0QH, UK; 3 Medical Research Council Rosalind Franklin Centre for Genomics Research, Hinxton, Cambridge CB10 1SB, UK

**Keywords:** CELLBIO, SIGNALING, PROTEINS

## Abstract

SNAREs provide the specificity and energy for the fusion of vesicles with their target membrane, but how they are sorted into the appropriate vesicles on post-Golgi trafficking pathways is largely unknown. We demonstrate that the clathrin-mediated endocytosis of the SNARE VAMP7 is directly mediated by Hrb, a clathrin adaptor and ArfGAP. Hrb wraps 20 residues of its unstructured C-terminal tail around the folded VAMP7 longin domain, demonstrating that unstructured regions of clathrin adaptors can select cargo. Disrupting this interaction by mutation of the VAMP7 longin domain or depletion of Hrb causes VAMP7 to accumulate on the cell's surface. However, the SNARE helix of VAMP7 binds back onto its longin domain, outcompeting Hrb for binding to the same groove and suggesting that Hrb-mediated endocytosis of VAMP7 occurs only when VAMP7 is incorporated into a cis-SNARE complex. These results elucidate the mechanism of retrieval of a postfusion SNARE complex in clathrin-coated vesicles.

## Introduction

Soluble N-ethylmaleimide-sensitive factor (NSF) attachment protein receptors (SNAREs) are type II transmembrane proteins that drive membrane fusion events on the secretory and endocytic pathways (Chen and Scheller, 2001). All SNAREs contain a 16-turn “SNARE” helix and can be defined as either Q- or R-SNAREs depending on the residue at layer 0 in the SNARE helix (Fasshauer et al., 1998). Only certain combinations of three Q-SNAREs from one membrane and one R-SNARE from the apposing membrane can interact to form a trans-SNARE complex that brings the membranes together to facilitate their fusion (McNew et al., 2000). The original model for SNARE distribution suggests that Q-SNAREs are in target membranes and their cognate R-SNAREs are in vesicle membranes. However, several studies now suggest that both Q- and R-SNAREs are found in both target and vesicle membranes (Mossessova et al., 2003; Pryor et al., 2004). Three important tasks must be achieved by the processes that mediate the sorting and trafficking of SNAREs. First, in order to ensure that two membranes fuse, sufficient amounts of the correct SNAREs must have been sorted into them. Second, for vesicle trafficking to continue in the cell and membrane identity to be maintained, some SNAREs must be recycled back to their steady-state locations for reuse, whereas others are retained in their current organelle membrane. Finally, any SNAREs that have become mislocalized from their normal trafficking routes need to be retrieved (Pelham, 2001).

The molecular mechanisms by which SNAREs are selected for incorporation into the different clathrin-coated vesicles (CCVs) that mediate many post-Golgi trafficking routes are largely unknown. Most do not possess the short linear motifs that are recognized by clathrin adaptors to direct the incorporation of standard cargo into CCVs, such as YxxΦ and [DE]xxxLL motifs (AP complexes), DxxLL motifs (GGAs), and FxNPxY motifs on LDL superfamily members (ARH and Dab2) (reviewed in Bonifacino and Traub, 2003). In addition, SNAREs are not known to be ubiquitinated, which would confer the ability to be bound by the epsins or GGAs, nor to have a docking surface for β-arrestins. However, many SNAREs possess a folded N-terminal domain of 100–150 residues that precedes the SNARE helical domain. These folded domains are either of unknown structure, such as those of Sec20 or Slt1, or defined as being a three-helical H_abc_ domain or a longin domain (Hong, 2005). Recent work (Miller et al., 2007) suggests the existence of processes that run in parallel to the short, linear motif recognition by general cargo/clathrin adaptors, whereby SNARE incorporation into CCVs is directed by the specific recognition of the folded N-terminal domains of SNAREs by other CCV components. Such a system would have the advantages that the transport of SNAREs would not be vulnerable to competition from standard motif-containing cargo, thus ensuring that sufficient amounts of the correct SNAREs are incorporated into a CCV, and at the same time it would not reduce the carrying capacity of CCVs for standard cargo.

Vesicle-associated membrane protein 7 (VAMP7), also known as tetanus neurotoxin-insensitive VAMP (TI-VAMP) or synaptobrevin-like 1 (SYBL1), is an R-SNARE that has been implicated in several fusion events, some likely to be in all cells and others occurring at specialized sites in particular types of differentiated cells. VAMP7 is the required R-SNARE for heterotypic fusion of lysosomes with late endosomes and also for fusion of lysosomes or vesicles with the plasma membrane (reviewed in Luzio et al., 2007). VAMP7 and yeast Nyv1p (Wen et al., 2006), along with the yeast and mammalian orthologs of Sec22 (Gonzalez et al., 2001) and Ykt6 (Tochio et al., 2001), comprise the “longin” subfamily of R-SNAREs because they possess a 120–140 residue N-terminal, profilin-like, longin domain (Filippini et al., 2001) that is also found in other proteins including the σ and μ subunits of the AP2 clathrin adaptor complex (Collins et al., 2002) and the SRα subunit of the signal recognition particle receptor (Schwartz and Blobel, 2003). Longin domains have been suggested to regulate trans-SNARE complex formation, possibly by folding over their SNARE helix (Gonzalez et al., 2001; Mancias and Goldberg, 2007; Martinez-Arca et al., 2000, 2003b; Tochio et al., 2001). In addition to its putative role in regulating trans-SNARE complex formation, the longin domain of VAMP7 has also been shown to be required for its sorting and delivery to late endocytic organelles by binding to the δ subunit of AP3 (Martinez-Arca et al., 2003b), for which it does not use a standard cargo trafficking YxxΦ or [DE]xxxLL motif.

VAMP7 must possess a complex, post-Golgi membrane traffic itinerary because it is involved in several different membrane fusion events. Because the coat composition of the transport vesicles mediating different steps in this itinerary will be different, it seemed likely that VAMP7 would have more than one vesicle-component binding partner. An obvious candidate for the location of binding sites on VAMP7 for membrane traffic machinery is the longin domain, especially given the observation that AP3 binds to this domain (Martinez-Arca et al., 2003a, 2003b), as well as the demonstration that the regulatory H_abc_ domain of the SNARE vti1b binds to the clathrin adaptor epsinR (Hirst et al., 2004; Miller et al., 2007). Consequently, a yeast two-hybrid cDNA library screen was performed with the VAMP7 longin domain in an attempt to identify new candidate binding partners that could act as cargo-selective devices for VAMP7. In this study, HIV Rev-binding protein (Hrb), also known as Rev-interacting protein (RIP) or Rev/Rex activation-domain-binding protein (Fritz et al., 1995), which is an ArfGAP (GTPase-activating protein), was identified as a binding partner of the longin domain of VAMP7. Hrb is shown to interact with AP2 and clathrin, and thus it could act as a highly specific cargo selective device for the clathrin-mediated endocytosis of VAMP7. The structure of the complex between the VAMP7 longin domain and the relevant portion of Hrb was determined and used to identify mutations that abolish the interaction. These mutations were subsequently used to confirm that in vivo, the VAMP7 longin domain/Hrb interaction is responsible for the efficient internalization of VAMP7 from the cell's limiting membrane.

## Results

### The VAMP7 Longin Domain Binds Hrb

**Figure 1:**
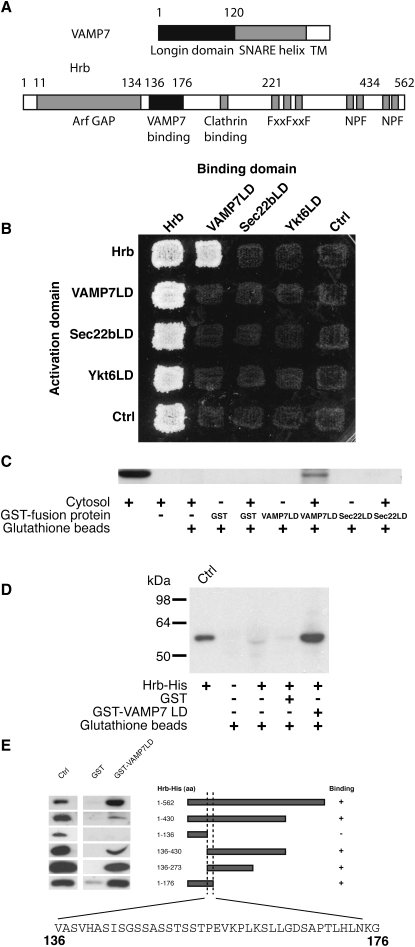
Identifying Hrb as a Binding Partner for VAMP7 (A) Schematic representations of VAMP7 and Hrb. VAMP7 contains a putative longin domain and a SNARE helix preceding its transmembrane helix (TM). Hrb has a predicted ArfGAP domain at its N terminus with the remainder of the protein predicted to possess very little secondary structure. This region contains a predicted clathrin-binding motif, three FXXFXXF motifs predicted to bind to AP2 appendage domains, and four NPF motifs that are able to bind EH domain-containing proteins. The full sequence of Hrb is shown in Figure S1A. (B) Yeast two-hybrid analysis indicates that VAMP7 longin domain (LD) but not Sec22b or Ykt6 longin domains (LDs) interacts with full-length Hrb, as indicated by growth of diploid strains containing the indicated plasmids on medium lacking adenine. The Hrb-DNA binding-domain construct autoactivates the reporter. Ctrl, empty binding- and activation-domain plasmids. (C) GST pull-down experiments indicate that VAMP7 longin domain but not Sec22b longin domain or GST alone binds full-length Hrb from A431 cell cytosol shown by immunoblotting with an anti-Hrb antibody. (D) GST-VAMP7 longin domain but not GST pulls down His_6_-tagged Hrb as indicated by immunoblotting with an anti-His_6_ antibody. (E) The binding of various C-terminally His_6_-tagged Hrb constructs to GST-VAMP7 longin domain demonstrates that the Hrb binding site for VAMP7 longin domain is located between residues 136 and 176.

To identify proteins that bind the longin domain of VAMP7, a yeast two-hybrid cDNA library screen using the longin domain as the bait protein was carried out. In this screen, 37 out of 39 positive clones encoded part of the AP3 complex δ subunit trunk domain. This agreed with the observations of Martinez-Arca et al. (2003b), who had previously demonstrated that AP3 is required for the correct localization of VAMP7. A single clone was identified whose cDNA encoded part of the ArfGAP Hrb (Fritz et al., 1995) (NCBI accession number NM_004504, shown schematically in Figure 1A; see full sequence in Figure S1A available online). To test the specificity of the interaction between the longin domain of VAMP7 and Hrb, we first used the yeast two-hybrid system to investigate the binding of full-length Hrb to the longin domains of VAMP7, Sec22b, and Ykt6. Strong yeast growth was only seen as a result of the interaction between Hrb and the longin domain of VAMP7, and there was no evidence of interaction with the longin domains of Sec22b and Ykt6 (Figure 1B).

To confirm the interaction between the VAMP7 longin domain and Hrb, bacterially expressed GST-VAMP7 longin domain was used in pull-down assays from A431 cytosol. Western blotting showed that GST-VAMP7 longin domain, but not GST-Sec22b longin domain nor GST alone, bound to endogenous Hrb (Figure 1C). Further binding studies using GST-VAMP7 longin domain showed that it bound to bacterially expressed His_6_-tagged Hrb (Figure 1D). A series of truncated, C-terminally His_6_-tagged Hrb constructs (Figure 1E) indicated that the sequence of Hrb that bound VAMP7 longin domain was between residues 136 and 176. This sequence is contained within the region that follows the ArfGAP domain, which is predicted to lack stable secondary structural elements (Cuff et al., 1998), and contains a number of motifs commonly found in endocytic clathrin adaptors. These include NPF and FXXFXXF, motifs which should bind EH domains and AP appendage domains, respectively, as well as a probable clathrin-binding motif (Figures 1A and S1A).

### Hrb Is an Endocytic CCV Component

**Figure 2:**
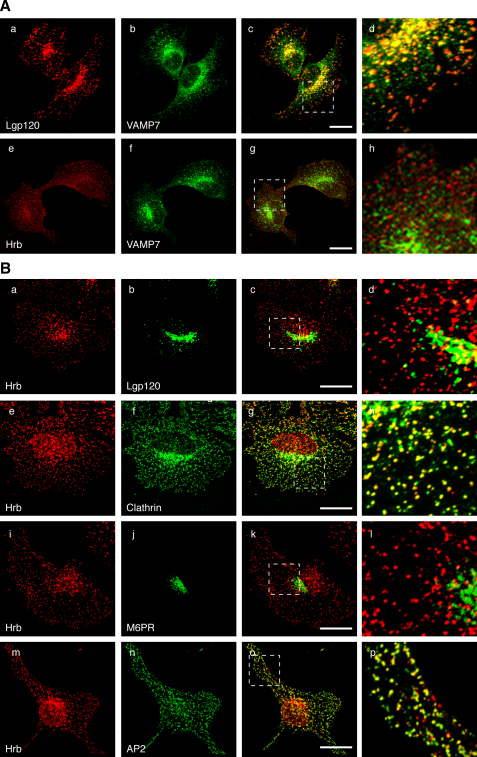
In Vivo Localization of VAMP7 and Hrb (A) NRK cells were double labeled with anti-lgp120 (a), anti-Hrb (e), or anti-VAMP7 (b and f) followed by fluorescently labeled secondary antibodies. Panels c and g are merged images of panels a and b and panels e and f, respectively. Panels d and h are higher magnifications of the regions boxed in panels c and g. (B) NRK cells double labeled with goat polyclonal anti-Hrb (panels a, e, i, and m) and mouse anti-lgp120 (b), mouse anti-clathrin (f), rabbit anti-MPR (j), and mouse anti-α-adaptin (n) followed by fluorescently labeled secondary antibodies indicate that Hrb is mainly found in plasma membrane-associated CCPs/CCVs. Panels c, g, k, and o are merged images and panels d, h, l, and p are higher magnifications of the regions boxed in panels c, g, k, and o. The higher-magnification images in panels h and p were analyzed using ImageJ software with a Pearson-Spearman correlation colocalization plug-in to perform quantitative statistical colocalization on the two-color confocal images as described by French et al. (2008). In both images, strong positive correlations for colocalization were indicated by r_P_ values of 0.51 (h) and 0.62 (p), and r_S_ values of 0.49 (h) and 0.57 (p). All images are confocal maximum-intensity z projections. The scale bars represent 10 μm.

Although the VAMP7 longin domain bound to Hrb in yeast two-hybrid and pull-down experiments, we found that VAMP7 and Hrb showed little, if any, colocalization by immunofluorescence confocal microscopy in cultured NRK (Figure 2A) or HeLa cells (data not shown). In agreement with previous studies (Martinez-Arca et al., 2003b; Pryor et al., 2004), we observed considerable overlap of VAMP7 localization with the lysosomal membrane glycoprotein lgp120 (Figure 2A). In contrast, membrane-associated Hrb showed no colocalization with either lgp120 or with the late endosomal marker cation-independent mannose 6-phosphate receptor (M6PR) in NRK cells (Figure 2B). However, Hrb did show extensive colocalization with clathrin and AP2 in clathrin-coated pits (CCPs) and CCVs (Figure 2B). These observations suggested that Hrb functions primarily at the plasma membrane in CCVs, consistent with the presence of a clathrin-binding motif (Figure S1), three FXXFXXF sequences that contain AP2 appendage-binding capability (221FANFAHF227, 240FANFDAF246, and 272FAHFDNF288) (Figure S1) (Edeling et al., 2006; Praefcke et al., 2004; Schmid et al., 2006), and NPF motifs that bind proteins containing EH domains including Eps15 and Eps15R (Doria et al., 1999).

### Structure Determination of VAMP7 Longin Domain/Hrb_136–176_ Complex

**Figure 3:**
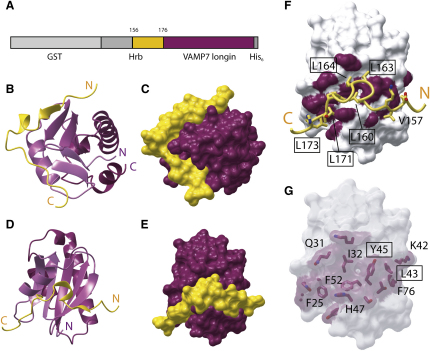
Structure of the VAMP7 Longin Domain/Hrb_136–176_ Complex (A) Schematic representation of the construct used to produce crystallizable VAMP7 longin/Hrb_136–176_. The GST tag, which is proteolytically cleaved off, and regions of the protein not visible in the electron density are colored gray. Those parts visible in the structure are colored as in (B) and (C), with VAMP7 in purple and Hrb in gold. (B–E) Structure of Hrb_136–176_ VAMP7 longin domain in ribbon and surface representations with VAMP7 in purple and Hrb in gold with (B) and (C), and (D) and (E), in the same orientations. (F) VAMP7 longin domain residues involved in Hrb binding are shown in purple on a surface representation with a Hrb fragment superimposed. Side chains that are important in binding to VAMP7 longin domain are indicated. (G) The same view as in (F) with Hrb removed. The side chains of VAMP7 involved in Hrb binding can be seen through the semitransparent VAMP7 surface.

Using residues 1–176 of Hrb and the VAMP7 longin domain in isothermal titration calorimetry (ITC), the K_D_ for the interaction was measured as 10.5 μM (see below). In order to increase artificially the apparent concentration of the two components and thus make crystallizing a complex of the two more likely, a cDNA encoding the fragment of Hrb mapped as binding VAMP7 longin domain (residues 136–176) was cloned onto the 5′ end of the VAMP7 longin domain cDNA. The Hrb sequence in the resultant chimeric protein was preceded by a cleavable GST tag, and a His_6_ tag was placed after the C terminus of VAMP7 longin domain (GST-Hrb_136–176_/VAMP7 LDHis_6_). Following removal of the GST tag, this chimeric construct was crystallized and its structure was determined by SIRAS with a single mercury derivative (Experimental Procedures), because attempts to solve it by molecular replacement using a variety of profilin-like fold proteins as search models failed. The chimeric construct crystallized with four molecules in the asymmetric unit (Figure S2A), with the fragments of Hrb binding to each longin domain in an identical manner but with two being intermolecular and two being intramolecular interactions (Figure S2). As expected, the VAMP7 longin domain (residues 1–115) possessed a five-stranded antiparallel β sheet, flanked by helix α1 on one face with helix α2 and a truncated helix α3 on the other (Figure 3). The first 20 residues of the Hrb fragment (residues 136–155) are disordered, and only residues 156–176 are visible in the density. These sit in a hydrophobic groove that wraps halfway around the longin domain (Figure 3) and have assumed short stretches of defined secondary structure on binding the longin domain. Residues 156–159 of Hrb form an additional β strand along one edge of VAMP7 longin domain strand β3. The Hrb short β strand then gives way to a single-turn α helix (residues 160–167) that is flanked by proline residues, and contacts the longin domain via hydrophobic interactions using predominantly three leucine residues. Residues 168–176 of Hrb pack against helix α1 of VAMP7 longin domain, with the interaction again being mediated by hydrophobic side chains and also one hydrogen bond between Asn24 of VAMP7 longin domain and Thr170 of Hrb. Overall, the interaction buries 900 Å^2^ of the solvent-accessible surface of VAMP7 longin domain and 1020 Å^2^ of Hrb, giving a total buried surface of 1920 Å^2^, and involves 13 residues from Hrb and 16 residues on VAMP7 longin domain and shows good shape complementarity (interface analyzed using PISA; Krissinel and Henrick, 2005) (see Figure 3).

**Figure 4:**
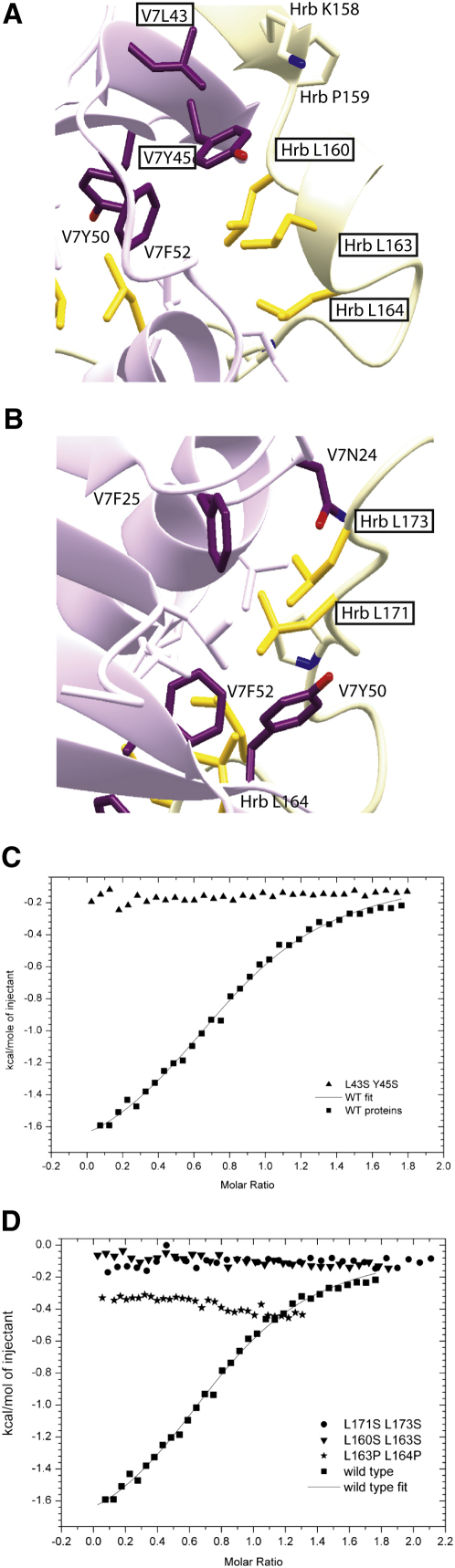
Mutation of Key Residues in the VAMP7 Longin Domain/Hrb Interface Inhibits Their Binding (A and B) Details of side chains involved in the interaction between VAMP7 longin domain and Hrb, with carbon atoms and ribbons colored as in Figure 3. Mutated residues studied in (C) and (D) are boxed. (C and D) The inhibitory effects of indicated mutations on VAMP7/Hrb binding as shown by ITC. The K_D_ for the interaction increases from approximately 10 μM to unmeasurable levels > 300 μM for the four mutations tested (see also Figure S2).

In order to confirm the validity of the interaction, mutations were made in pairs of key residues involved in the interaction (N24A/F25S, L43S/Y45S, and Y50S/F52S in the context of GST-VAMP7 longin domain and L160S/L163S, L163P/L164P, and L171S/L173S in the context of Hrb_1–176_ His_6_) (Figures 4A and 4B). The mutations N24A/F25S and Y50S/F52S in VAMP7 longin domain resulted in the protein being expressed in an insoluble form. However, L43S/Y45S in VAMP7 longin domain and the three mutations in Hrb were soluble and did not alter the fold of the proteins, as estimated by circular dichroism (data not shown). ITC (Figures 4C and 4D) and GST pull-down (Figures S2D and S2E) experiments showed that, as predicted, all the soluble mutations strongly inhibited the interaction between the two proteins, with the K_D_ for the binding increasing from around 10 μM for wild-type to undetectable levels (values of >300 μM).

### Hrb Functions as an Endocytic Adaptor for VAMP7 at the Cell Surface

**Figure 5:**
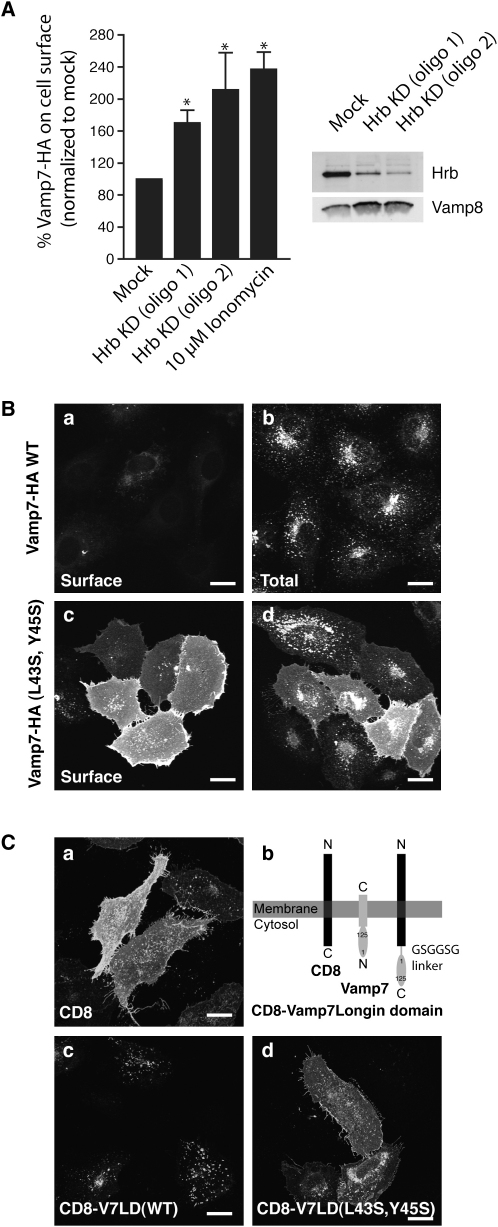
Effect of Disrupting the VAMP7 Longin Domain/Hrb Interaction In Vivo (A) NRK cells stably expressing VAMP7-HA transfected with siRNA against Hrb (oligos 1 and 2) showed an increase in VAMP7-HA at the cell surface that was not observed following mock transfection (see also Figure S3A). Cells were fixed with paraformaldehyde, and an aliquot was permeabilized with Triton X-100. Cells were then incubated with [^125^I]mouse monoclonal anti-HA. For each cell preparation the amount of VAMP7-HA on the cell surface, as a percentage of the total amount of VAMP7-HA expressed, was calculated (histogram, n = 3 ± SEM; ^∗^p < 0.05 versus mock). A representative western blot shows the levels of Hrb in mock and Hrb knockdown (KD) cells. The VAMP8 blot is a control for protein loading. (B) NRK cells stably transfected with ΔpMEP4 containing either wild-type (WT) (a and b) or L43S/Y45S mutant (c and d) VAMP7-HA were treated with CdCl_2_ to induce expression. They were then either fixed with paraformaldehyde (for surface staining of HA [a and c]) or fixed and then permeabilized with Triton X-100 (for total staining of HA [b and d]). For an expanded figure, see Figure S6A. Cells stably expressing L43S/Y45S mutant VAMP7-HA have increased VAMP7-HA at the cell surface. The total amount of WT or L43S/Y45S mutant VAMP7-HA expressed in the transfected cells was the same (Figure S6B), but the amount of HA tag was reduced when the WT construct was exposed to the proteolytic environment of lysosomes. (C) NRK cells transiently expressing chimeric ΔpMEP4 constructs encoding CD8 (a) or CD8 followed by a flexible linker consisting of amino acids GSGGSG (as depicted in [b]) connecting it to either the wild-type (c) or L43S/Y45S mutant (d) longin domains. For an expanded figure, see Figure S7. The amount of CD8 on the surface and in internal structures was visualized by immunofluorescence using mouse anti-CD8. Images are confocal maximum-intensity z projections. The scale bars represent 20 μm.

To investigate the possibility that Hrb functions as an endocytic adaptor for VAMP7, we knocked both Hrb and clathrin down using siRNAs. NRK cells expressing stable full-length human VAMP7 tagged with the HA epitope at the C terminus under an inducible promoter were treated with two separate siRNA oligonucleotide pairs (oligos 1 and 2) against Hrb and chc-2 (Supplemental Data) against clathrin. Depletion of Hrb resulted in an increased amount of VAMP7-HA on the cell surface of NRK cells when compared with mock transfected cells (Figure S3). Quantification using an ^125^I-labeled monoclonal anti-HA antibody indicated that there was a doubling of VAMP7-HA on the cell surface following ∼90% depletion of Hrb (Figure 5A), similar to that demonstrated following addition of ionomycin (Figure 5A), a treatment that results in lysosome fusion with the plasma membrane and the consequent appearance at the cell surface of lysosomal membrane proteins (Figure S3B) (Rodriguez et al., 1997). Depletion of clathrin also caused a marked increase in the level of VAMP7-HA on the plasma membrane (Figure S4). Consistent with Hrb functioning as a SNARE-specific adaptor, depleting HeLaM cells of Hrb by siRNA had no effect on the internalization and intracellular accumulation of EGF nor on its degradation when compared to mock transfected cells (Figure S5).

The observation that recombinant VAMP7 longin domain mutated at L43S/Y45S folded correctly but failed to bind Hrb suggested a further experiment to confirm the function of Hrb as an endocytic adaptor for VAMP7. Whereas in NRK cells stably transfected with wild-type VAMP7-HA the tagged protein was mainly in lysosomes with very little on the cell surface, cells transfected with the L43S/Y45S mutant of VAMP7-HA showed a marked increase in cell-surface localization of tagged protein (Figures 5B and S6A), despite expression levels of VAMP7 being equivalent (Figure S6B). Analogous results were obtained when the experiment was performed in the absence of endogenous VAMP7, whose level had been depleted by siRNA treatment (data not shown). Further, when the longin domain of VAMP7 was attached via a linker (GSGGSG) to the neutral reporter CD8 (Figure 5C), the resulting construct was efficiently localized to lgp110-positive late endosomes/lysosomes. However, when the L43S/Y45S non-Hrb-binding mutant version of the longin domain was fused to CD8, this construct accumulated on the plasma membrane (Figure 5C, panel d; Figure S7). Taken together, these experiments demonstrate that Hrb functions as a cargo/clathrin adaptor in clathrin-mediated endocytosis of VAMP7 through the interaction of the VAMP7 longin domain with Hrb.

### Hrb Competes for the SNARE Helix Binding Site on VAMP7

**Figure 6:**
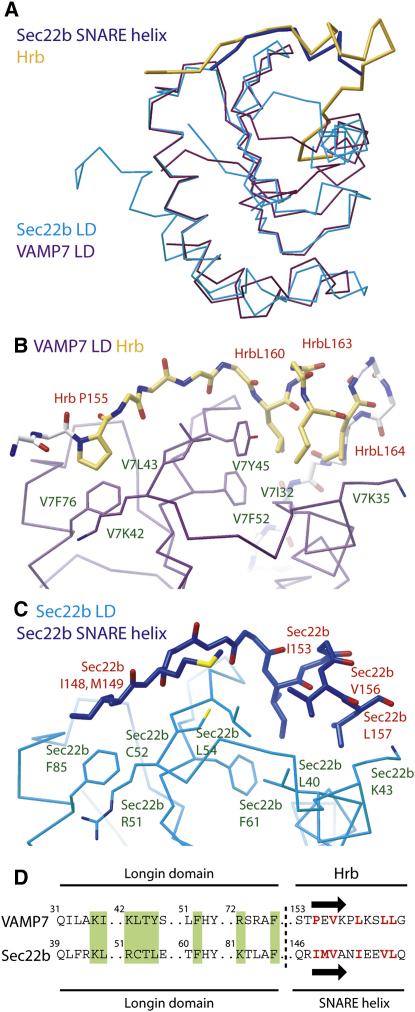
VAMP Analogous Binding Sites Exist on VAMP7 and Sec22b Longin Domains (A) Superposition of Cα traces of the longin domains of VAMP7 (purple) and Sec22b (cyan) with the Hrb peptide (gold) and Sec22b SNARE peptide (dark blue) can be seen to have the same conformations and superimpose well. (B and C) Enlargements of the binding sites showing molecular detail for the hydrophobic side-chain interactions that mediate the binding of VAMP7 LD/Hrb peptide and Sec22b LD/Sec22b SNARE peptide colored as in (A). (D) Structure-based alignment of the longin domains of Sec22b and VAMP7 and their respective binding partners the intramolecular SNARE helix of Sec22b and HrbVAMP. Key residues on the longin domains involved in their interactions are boxed in green and varied numbers of intervening residues are indicated by dots. Key residues on Hrb and the Sec22b SNARE helix along with those proposed to be important for VAMP7 SNARE helix binding are colored red. The arrows indicate the short β strand in the two determined structures.

**Figure 7:**
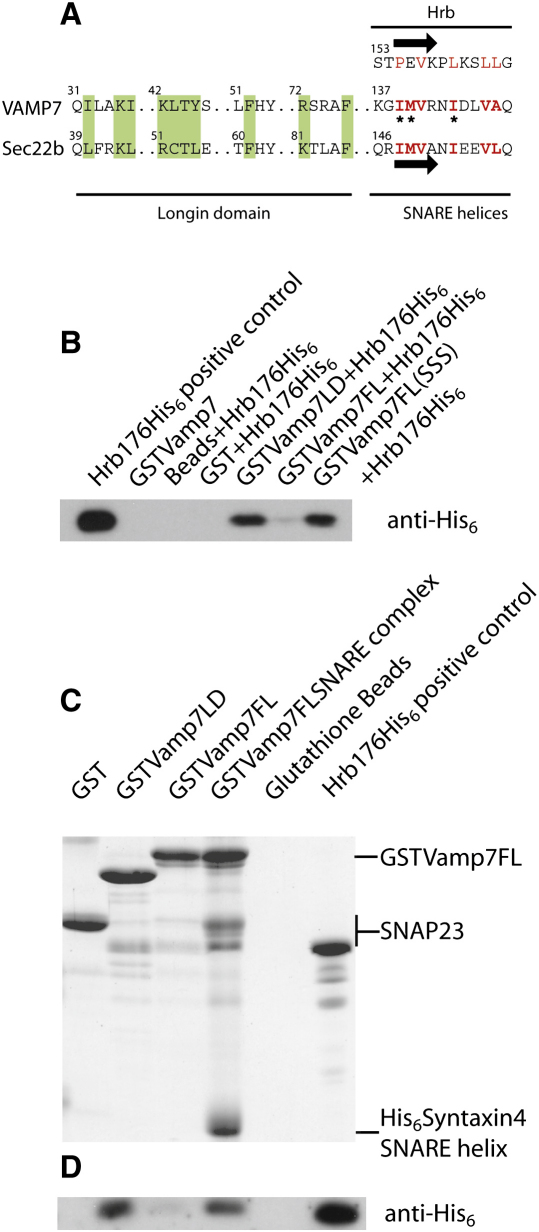
Hrb Competes for the SNARE Helix Binding Site on VAMP7 Longin Domain (A) Structure-based alignment as in Figure 6D with the putative “bind-back” sequence of VAMP7 also included. Residues mutated to abolish the proposed interaction of VAMP7 longin domain with its SNARE helix are indicated by an asterisk. (B) Western blotting with anti-His_6_ antibody of GST pull-down experiment for the interaction of various VAMP7 constructs with Hrb_1–176_ His_6_. The GST-VAMP7 longin domain (LD) and the GST-VAMP7 whole cytoplasmic domain (I139S/M140S/I144S) triple mutant (GST-VAMP7FL[SSS]) bound Hrb, but the wild-type construct (GST-VAMP7FL) did not. (C and D) Coomassie blue staining (C) and western blotting with anti-His_6_ antibody (D) of GST pull-down experiment for the interaction of VAMP7 longin domain, and the whole cytoplasmic domain of VAMP7 on its own and in an endocytic SNARE complex, with His_6_syntaxin4 (201–266) and SNAP23 (1–210). As in (B), the longin domain of VAMP7 bound Hrb_1–176_ His_6_, whereas the full-length cytoplasmic domain of VAMP7 did not. Formation of a VAMP7/SNARE complex restored Hrb_1–176_ His_6_ binding to the levels displayed by the longin domain alone.

It has been shown that in order for Sec22b to bind to the Sec23/24 component of the COPII coat, both its longin domain and an intact SNARE helix are required (Liu et al., 2004; Mancias and Goldberg, 2007; Mossessova et al., 2003). The recently determined structure of the Sec22b longin domain complexed with Sec23/24 (Mancias and Goldberg, 2007) showed that a small part of the Sec22b SNARE helix binds back onto and stabilizes its longin domain such that it can interact with Sec23/24. Binding back and interaction with Sec23/24 would be impossible if the Sec22b SNARE helix was participating in a cis-SNARE complex, which leads to the conclusion that Sec23/24 can only bind to isolated, that is, uncomplexed, Sec22b. Overlaying the structures of the VAMP7 longin domain and Sec22b longin domain (Figure 6A) shows that the two possess a very similar hydrophobic groove that binds the first half of the Hrb fragment in the case of VAMP7 and binds back on the SNARE helix in the case of Sec22b, both in very similar conformations and using very similar, mainly hydrophobic, side chains (Figures 6B and 6C). However, the region of the Sec22b longin domain analogous to the site on the VAMP7 longin domain where residues 168–176 of the Hrb sequence bind is different in the two proteins, explaining why Hrb cannot bind Sec22b (Figure 1). Comparison of the SNARE helix sequences of VAMP7 and Sec22b reveals a high degree of similarity in the region that in Sec22b binds back on its longin domain and, indeed, the major determinants of the Sec22b SNARE helix binding Ile139, Met140, and Ile144 (numbering as in VAMP7) are absolutely conserved between the two proteins (Figure 6). These residues also align well with the residues in Hrb bound by VAMP7 longin domain (Figure 6). These observations suggested that the SNARE helix of VAMP7 also binds back onto its longin domain, a suggestion that is in line with Martinez-Arca et al. (2003b). However, because the SNARE helix binding back to the longin domain would occur at the same site as Hrb binding, they would be mutually exclusive. Further, because binding back of the VAMP7 SNARE helix onto its longin domain is intramolecular as opposed to Hrb binding which is intermolecular, the former would be much stronger and predominate in solution. To test the proposal that Hrb and the SNARE helix of VAMP7 compete for the same site on the VAMP7 longin domain, GST pull-down experiments were performed using GST-VAMP longin domain alone, GST-VAMP7 full-length wild-type cytoplasmic domain, and a fully folded (as determined by circular dichroism; data not shown) mutant version of the latter in which the key binding Ile139, Met140, and Ile144 residues were mutated to serines (Figure 7A). Figure 7B shows that VAMP7 longin domain alone effectively bound Hrb, but full-length, wild-type cytoplasmic domain of VAMP7 demonstrated very little binding. However, mutation of the proposed key longin domain-binding residues in the SNARE helix of VAMP7 restored Hrb binding. In vivo, the sequestration of this “bind-back” sequence in VAMP7 would occur on SNARE complex formation, which at the plasma membrane would likely be with SNAP23 and syntaxin4 (Rao et al., 2004). Consequently, a 1:1:1 stoichiometric complex was made between recombinant, bacterially produced, full-length VAMP7 cytoplasmic domain, SNAP23, and the SNARE helix of syntaxin4 and its ability to bind Hrb compared with that of both the isolated VAMP7 longin domain and full-length cytoplasmic domain. As shown in Figures 7C and 7D, whereas isolated full-length VAMP7 showed little binding to Hrb, participation in a SNARE complex restored Hrb binding of the VAMP7 cytoplasmic domain to levels comparable to that displayed by the isolated longin domain.

## Discussion

The correct complement of SNARE proteins helps to define a specific organelle membrane and plays a major role in determining the specificity of protein sorting by permitting fusion of transport vesicles with only certain target membranes (McNew et al., 2000; Pelham, 2001). To date, all lysosomal fusion events in which the trans-SNARE complex has been defined appear to require the R-SNARE VAMP7 (reviewed in Luzio et al., 2007), and thus the high concentration of VAMP7 on the lysosome membrane may help to define the lysosome. The retrieval of VAMP7 from the plasma membrane is necessary for its use in subsequent endosomal/lysosomal fusion events, but may also prevent inappropriate endocytic organelle fusion with the plasma membrane. Our data indicate that the clathrin-mediated endocytic retrieval of VAMP7 from the cell surface is mediated by the ArfGAP Hrb, which possesses all the properties of a cargo-binding clathrin adaptor in that it can be recruited into forming CCVs by its interactions with AP2 appendages and clathrin (Figure S1 and Schmid and McMahon, 2007). It will also be concentrated in coated pits through its interactions with EH domain-containing proteins (Doria et al., 1999) and very likely a membrane-bound Arf while also binding to its cargo VAMP7. Under physiological circumstances, VAMP7 would become localized to the cell's limiting membrane following fusion of late endocytic compartments, or vesicles derived from them, during processes including lysosome exocytosis, membrane repair, and neurite outgrowth (see Luzio et al., 2007 for references). Given the presence of AP3, whose δ subunit can also bind VAMP7 longin domain, on the recycling endosome (Peden et al., 2004), we expect that AP3 can drive the subsequent sorting of VAMP7 to late endosomes/lysosomes (Martinez-Arca et al., 2003b) following internalization from the cell surface. A schematic representation of this pathway of retrieval of VAMP7 from the plasma membrane is shown in Figure S8.

It seems probable that VAMP7 is retrieved from the plasma membrane not as an isolated SNARE but as part of a heterotetrameric cis-SNARE complex, presumably with SNAP23 and syntaxin4 (Rao et al., 2004) left there following lysosome fusion. This conclusion follows from our observation that in VAMP7 the SNARE helix interaction with the longin domain is intramolecular and would thus outcompete the intermolecular Hrb interaction for longin domain binding unless this competition is relieved by sequestration of the VAMP7 SNARE helix as part of a higher-affinity SNARE complex (Yang et al., 1999). This is the opposite situation to that in the incorporation of Sec22b into COPII vesicles, where binding to the Sec23/24 complex only occurs when the Sec22b SNARE helix does bind intramolecularly to the Sec22b longin domain, so that Sec22b can only be trafficked by this mechanism when it is free of its SNARE complex partners (Liu et al., 2004; Mancias and Goldberg, 2007). These opposite effects of sorting on either complexed or free SNAREs, respectively, arise as a direct result of VAMP7 using the same site for cargo selection and SNARE helix binding, whereas Sec22b uses different but positive allosteric sites for the two binding functions. The conclusion that VAMP7 must be endocytosed as part of a cis-SNARE complex further suggests that the dissociation of the VAMP7-containing SNARE complex by α-SNAP and NSF must occur after the endocytic event.

In common with other integral membrane cargo, the intracellular localization of SNAREs is determined by delivery, retrieval, and retention mechanisms. The interaction of cargo with a coat component, however, needs to be of moderate affinity (1–50 μM K_D_) and therefore dynamic, in order that the proofreading of cargo incorporated into CCVs and the dissociation of cargo and adaptors during clathrin cage disassembly can readily occur. The 10.5 μM K_D_ interaction between the surface patch on VAMP7 longin domain and a portion of the Hrb unstructured C-terminal domain must be highly specific to these two components, because the topography of the VAMP7 longin domain surface is dependent on its overall fold, and the sequence of the Hrb-binding fragment is not found in other clathrin adaptors. This study therefore shows how a specific SNARE, in this case most probably present as a SNARE complex, can be sorted into a single type of CCV through the interaction of its folded N-terminal domain with a CCV component in a manner that meets the required kinetic and thermodynamic parameters required for dynamic cargo selection (reviewed in Bonifacino and Traub, 2003; Edeling et al., 2006).

In addition to the widely used recognition by clathrin adaptors of generic transplantable motifs, or covalently attached ubiquitin selecting cargo for incorporation into CCVs, the evidence presented here demonstrates that highly specific interactions unique to a given SNARE/clathrin adaptor pair can also be used to sort SNAREs into CCVs. These CCV components can, as is demonstrated by Hrb's role as an ArfGAP, have other roles in CCV formation. By working in parallel with, but not competing with, the incorporation of standard motif-containing cargo, such mechanisms of SNARE folded domain recognition will ensure that the desired SNARE(s) is efficiently incorporated into CCVs, while the carrying capacity for standard motif-containing cargo is not reduced. When taken in conjunction with data on the interaction of vti1b with epsinR (Miller et al., 2007) and of VAMP7 with AP3 (Martinez-Arca et al., 2003b), the data shown here provide compelling evidence that the use of folded “regulatory” domain-mediated, rather than of the commonly used short, linear motif-mediated interactions, is the paradigm for sorting SNAREs into post-Golgi transport vesicles. The recent demonstration that Sec22b also uses its longin domain to bind to components of the COPII vesicle coat (Mancias and Goldberg, 2007) lends yet further support to this notion.

Finally, the discovery that the predicted unstructured region of the clathrin adaptor Hrb can act directly in cargo selection, where the cargo recognition determinant, the longin domain of VAMP7, has significant tertiary structure, demonstrates both a new mechanism for CCV cargo recognition and a new role for the unstructured regions of clathrin adaptors that were previously believed to be involved only in their recruitment to and maintenance in CCVs (Schmid and McMahon, 2007). Thus, the whole sequence of clathrin adaptors, rather than just their folded domains, should be considered when searching for the binding site of a given cargo molecule.

## Experimental Procedures

Vectors were constructed from the cDNAs encoding full-length human VAMP7, human Hrb, human HrbL, mouse Sec22b, and mouse Ykt6 (IMAGE clones 6503665, 4836885, 4810320, 5119580, and 5370491, respectively) and mouse VAMP7 longin domain, which was a gift from Rob Piper (University of Iowa). Constructs used in this study are detailed in Supplemental Data.

### Yeast Two-Hybrid Library Screening

Yeast strain PJ69-4A (*MAT*α) was transformed with the bait plasmid pGBDU-C1 (James et al., 1996) encoding the longin domain (1–120) of VAMP7 and screened against a K562 erythroleukemia cDNA Matchmaker library (BD Biosciences) as previously described (Lehner et al., 2004). Identification of interacting proteins by plasmid sequencing and BLAST searching was as previously described (Reid et al., 2005). In order to confirm the interaction with the identified prey DNA (Hrb), the longin domains of VAMP7, Sec22b (residues 1–127), Ykt6 (residues 1–132), and Hrb were all cloned into both *GAL4*-DNA binding-domain and *GAL4*-DNA activation-domain plasmids, transformed into yeast strain PJ69-4A (*MAT***a** for activation-domain plasmids and *MAT*α for binding-domain plasmids), and longin domain/Hrb interactions tested in yeast two-hybrid analysis. Interactions between binding- and activation-domain fusion proteins were scored by growth of the yeast as in Bowers et al. (2004).

### Protein Expression, Purification, and Crystallization

All recombinant proteins were expressed in BL21(DE3)/pLysS cells for 16 hr at 22°C following induction with 0.2 mM IPTG at 37°C. Cells were lysed in 10 mM Tris-HCl (pH 8.5), 250 mM NaCl (buffer A), 1 mM DTT, and protease inhibitors and the protein affinity purified on glutathione-Sepharose or Ni^2+^-NTA-agarose where appropriate.

For GST-Hrb_136–176_/VAMP7 LDHis_6_ destined for crystallization, the GST tag was cleaved with thrombin overnight at room temperature while the protein was bound to the beads. The cleaved protein was eluted and the thrombin cleavage was halted by the addition of AEBSF. The eluted protein was then incubated with Ni^2+^-NTA-agarose for 1 hr at 4°C. The resin was pelleted, packed in a column, and washed with 0.5 L buffer A plus 20 mM imidazole and the protein was eluted with buffer A containing 250 mM imidazole. This protein, Hrb_136–176_/VAMP7 LDHis_6_, was further purified by S200 gel filtration in buffer A, and concentrated to 7.5 mg/ml. The best crystals grew by sitting-drop vapor diffusion against a reservoir containing 20% PEG6000, 100 mM citric acid (pH 4.0), and 0.2 M LiCl. Mercury derivative crystals were prepared by washing crystals in non-DTT-containing reservoir buffer and then soaking them for 10 min in reservoir buffer supplemented with 0.5 mM ethyl mercury thiosalicylate. All crystals were cryo-protected in 22% PEG6000, 100 mM citric acid (pH 4.0), 0.2 M LiCl, and 20% glycerol for data collection at 100K. Crystals were of space group P 2_1_2_1_2 with unit-cell dimensions a = 55.4 Å, b = 105.5 Å, c = 115.1 Å, α = β = γ = 90.0° and diffracted to 2.2 Å. The structure was solved by SIRAS with seven mercury sites. The final model of the asymmetric unit contained four molecules, two with an intermolecular interaction and two with an intramolecular interaction of the longin domain with a Hrb fragment, and was refined to final R_cryst_ and R_free_ values of 22.8% and 28.6%, respectively, with no residues in disallowed regions. A complete explanation of the structure determination and full statistics may be found in Supplemental Data.

### Protein/Protein Interaction Experiments

GST fusion proteins were eluted from glutathione-Sepharose with buffer containing 30 mM reduced glutathione and subsequently dialyzed back into buffer A. Where necessary, GST tags were removed as described above.

GST pull-down protein/protein interaction experiments were carried out using appropriate GST-tagged bait proteins and either His_6_-tagged prey proteins in either PBS or 20 mM HEPES (pH 7.5), 200 mM NaCl supplemented with 0.1% (v/v) IGEPAL or cell lysates prepared as in Hirst et al. (2004). Binding experiments were carried out at 4°C for 1 hr. The supernatants were removed and the beads were washed three (His-tagged proteins) or five times (lysates) with 1 ml of buffer containing 0.1% (v/v) IGEPAL. Bound proteins were analyzed by SDS-PAGE and western blotting with appropriate antibodies.

For ITC experiments, all GST tags were removed. VAMP7 longin domain (1.4 ml) was placed in the cell at 0.15–0.2 mM, and Hrb at 1.5–2 mM was titrated in 30–40 injections of 4–8 μl. The heat of dilution of Hrb into buffer was subtracted from the data and titration curves were fitted using ORIGIN (http://www.originlab.com/), yielding values for the stoichiometry N, equilibrium association constant K_A_ (=K_D_^−1^), and enthalpy of binding.

The VAMP7 SNARE complex consisting of GST-VAMP7(1–188), His_6_syntaxin4 (201–266), and SNAP23 (made by eluting the products of thrombin cleavage of GSTSNAP23 that was bound to glutathione-agarose beads [1–210]) was made by incubating the proteins at 4°C for 48 hr in the molar ratio of 1:4:4 in 20 mM HEPES (pH 7.5), 200 mM NaCl, and 0.1% (v/v) IGEPAL. GST beads were added for 1 hr and the resulting stoichiometric 1:1:1 complex was washed three times on beads with 20 mM HEPES (pH 7.5), 200 mM NaCl, and 0.1% (v/v) IGEPAL. The stoichiometry of the complex was checked prior to use in pull-down experiments by densitometry of a Coomassie-stained SDS-PAGE gel as being 1:1:1. Binding to Hrb was then carried out as outlined above.

### Antibodies

Anti-Hrb goat polyclonal antibody (sc-1424) was from Santa Cruz Biotechnology. Anti-His_6_tag rabbit polyclonal antibody (ab9108), anti-α-adaptin monoclonal antibody (AP6), and anti-clathrin monoclonal antibody (X22) were from Abcam. Anti-CD8 monoclonal antibody (UCHT4) was from Ancell and anti-HA monoclonal antibody (HA.11) was from Covance. Anti-VAMP7 rabbit polyclonal antibody was raised against GST-VAMP7 longin domain (mouse VAMP7 amino acids 1–120) and affinity purified using His_6_-VAMP7 longin domain. Monoclonal anti-rat lgp120 antibody (GM10) and polyclonal anti-rat lgp110 antibody (580) were as described (Reaves et al., 1996).

### Cell Culture, Transfection, and Hrb Depletion

Details of mammalian cell culture, transfection methods, and siRNA treatment are given in Supplemental Data.

### Immunofluorescence

For immunofluorescence, cells were fixed with 4% (w/v) paraformaldehyde. To remove cytosolic, non-membrane-associated Hrb, cells were prepermeabilized with 0.05% (w/v) saponin in a cytosolic extraction buffer (25 mM HEPES-KOH [pH 7.4], 25 mM KCl, 2.5 mM Mg acetate, 5 mM EGTA, 150 mM K glutamate) for 1 min at 21°C before fixation. To permeabilize cells after fixation, all blocking, washing, and immunolabeling steps were performed in 0.05% (w/v) saponin in 0.2% (w/v) BSA in PBS. Immunolabeling of nonpermeabilized cells used the same PBS-BSA solution, but without the addition of the saponin. Alexa Fluor-labeled secondary antibodies were from Molecular Probes. Images were captured using a Zeiss LSM 510 confocal system on an Axiovert 200M microscope.

### Iodination of Monoclonal Anti-HA and Binding Studies

NRK cells stably expressing VAMP7-HA were fixed with paraformaldehyde and labeled with ^125^I-labeled monoclonal anti-HA (HA.11; Covance), with or without detergent-mediated permeabilization, to determine % cell-surface VAMP7-HA as described in Supplemental Data.

## Accession Numbers

Coordinates and structure factors have been deposited in the Protein Data Bank under ID code 2VX8.

## Supplemental Data

Document S1. Supplemental Experimental Procedures, One Table, Supplemental Discussion, and Nine Figures
