# Supplementary material for: Molecular Basis for the Sorting of the SNARE VAMP7 into Endocytic Clathrin-Coated Vesicles by the ArfGAP Hrb
Source: Cell. 2008 Sep 5;134(5):817–27. doi: 10.1016/j.cell.2008.07.023 (PMC2648964; doi:10.1016/j.cell.2008.07.023)
Supplement: Document S1. Supplemental Experimental Procedures, One Table, Supplemental Discussion, and Nine Figures [file mmc1.pdf]

Cell, Volume *134*

Supplemental Data

Molecular Basis for the Sorting of the SNARE VAMP7 into Endocytic Clathrin-Coated Vesicles by the ArfGAP Hrb

Paul R. Pryor, Lauren Jackson, Sally R. Gray, Melissa A. Edeling, Amanda Thompson, Christopher M. Sanderson, Philip R. Evans, David J. Owen, and J. Paul Luzio

Supplemental Experimental Procedures

Constructs used in this study

pGEXVAMP7 LD (1-120), pGEXSec22bLD (1-127), pGEXYkt6LD(1-132), pGEXHrb(136-176)VAMP7 LDHis6,

pGEX VAMP7 LDHis$_6$ (wt, N24A/F55S, L43S/Y45S, Y50S/F52S), pGEXHrb(1-176)His$_6$ (wt, L160S/L163S, L163P/L164P, L171S/L173S), pGEXHrb(1-562)His$_6$, pGEXHrb(1-430)His$_6$, pGEXHrb(1-136)His$_6$, pGEXHrb(136-430)His$_6$, pGEXHrb(136-273)His$_6$, pGEXHrb(1-290)His$_6$(wt and D286S/F288A), pGEXHrb(1-219)His$_6$, pGEXHrb(1-229)His$_6$(wt and F227A), pGEXHrb(1-248)His$_6$(wt and D244S/F246A) pGEXVAMP7(1-188) (wt and I139S/I140S/I144S), ΔpMEP4-VAMP7-HA (wt and L43S/Y45S). ΔpMEP4-CD8-VAMP7(1-125) (wt and L43S/Y45S), pMWHis$_6$syntaxin4(201-266), pGEXSNAP23(1-210), pEGFPHrbL

Cell Culture.

Normal Rat Kidney (NRK), HeLa and A431 cells were cultured in Dulbecco's modified Eagle's medium (DMEM) containing 10% (v/v) foetal calf serum (FCS), 2mM L-glutamine, 100U/ml penicillin, 0.1 mg/ml streptomycin in a 5% CO_2 humidified atmosphere.

NRK cell transfection.

NRK cells were transfected using Fugene 6 transfection reagent (Roche Diagnostics Ltd.) at a ratio of 2μg DNA: 6 μl Fugene. NRK cells were transfected with ΔpMEP4-VAMP7 constructs and stable cell lines generated by growing cells in DMEM supplemented with 0.2 mg/ml hygromycin (Roche Diagnostics Ltd.). ΔpMEP4 contains a metallothionein promoter, hence expression of VAMP7-HA (stable cell lines) or CD8 constructs (transients) was induced by adding 10 μM $CdCl_2$ for 16 h to the tissue culture medium before experiments, NB treatment with cadmium results in some variation of expression levels in individual cells. In the case of CD8 constructs 21μM leupeptin was added with the $CdCl_2$ to inhibit lysosomal proteases.

RNAi experiments.

dsRNA oligonucleotides were designed to the cDNA of rat Hrb. The oligonucleotide sense sequences were as follows

5'-GAUGGUAUGUUCCUCCAGAUU-3' (oligo 1),

5'-GUAAAGCUCCUGUUGGUUCUU-3' (oligo 2).

For depletion of clathrin a single oligonucleotide chc-2 as described in (Motley et al., 2003) was used. There is a single mis-match between this oligonucleotide and the rat sequence, but after a single transfection of siRNA at 10nM there was a decrease in clathrin heavy chain levels (Supplementary Figure S4).

The corresponding anti-sense sequences were phosphorylated at their 5' end. A single dsRNA oligonucleotide was also designed to human Hrb (used in EGF assays), the sense sequence was 5'-AGUCGUGGCAUCAGUUCAUdTdT-3'. The TSG101 and AMSH siRNA duplexes were as described (Garrus et al., 2001; McCullough et al., 2004). All dsRNA oligonucleotides were purchased from

Dharmacon. RNAi experiments were performed using oligofectamine and dsRNA oligonucleotides at 100 nM.

Iodination of monoclonal anti-HA and binding studies.

20μg of purified monoclonal anti-HA (clone HA.11; Covance) was iodinated for 10 min using ^{125}I by the Iodogen method (Pierce) and free iodide removed by passage through an Econo-Pac 10DG desalting column (Bio-Rad). A typical iodination resulted in a specific activity of ~5,000 Ci/mmole. For binding studies, NRK cells cells stably expressing VAMP7-HA, and un-transfected NRK cells, were fixed with 4% paraformaldehyde for 20 min at 21˚C. For total binding, half of the cells for each condition were permeabilised with 0.1% (v/v) Triton X-100 for 10 min at 21˚C. All cells were then incubated in 0.2% (w/v) BSA in PBS for 1 h at 37˚C. Cells were then incubated with the iodinated antibody (50 μCi/ml; 0.3ml/well of a 24 well dish) in PBS supplemented with 0.2% (w/v) BSA, for 1 h at 21˚C. Unbound antibody was removed by washing cells 8X with 1 ml of the PBS-BSA solution. Cells were solubilised with 200 μl of 0.2M NaOH, and the radioactivity counted. Total protein/well was also measured using the BCA protein assay (Pierce). Calculations of the % cell surface VAMP7-HA was determined by dividing the counts/μg protein for non-permeabilised cells by the counts/μg protein for permeabilised cells for each condition. Background counts were determined by using un-transfected NRK cells and background surface and total counts were taken into account in all calculations.

EGF uptake and degradation assays.

Epidermal growth factor (EGF) uptake and degradation was performed as previously described (Bowers et al., 2006). The graph in Supplementary Figure S4 represent counts in the TCA supernatants (*i.e.* degraded ^{125}I-EGF) per μg of protein in

each sample as a percentage of the total counts bound at time zero (also per µg of protein).

Structure Determination

Data were processed using MOSFLM (Leslie, 2006) and scaled with SCALA (Evans, 2006). The best data set for a native Hrb/V7LD crystal was collected at the ESRF on beamline 14-4, λ= 0.97930 Å. The crystals were of space group $P2_12_12$ with cell dimensions were a= 55.4 Å, b= 105.5 Å, c= 115.1 Å, α=β=γ= 90.0°. For the best mercury derivative, data were collected at the ESRF, beamline 23-1, λ=1.0 Å. The space group was again $P2_12_12$, and cell dimensions were a= 55.5, b= 105.4, c= 115.4, α=β=γ= 90°.

The structure was determined by SIRAS using one native and one mercury derivative data as molecular replacement techniques failed. Seven mercury sites were identified in the derivative using autoSHARP (Vonrhein et al., 2006), and phasing was subsequently undertaken with SHARP (Bricogne et al., 2003). Although the derivative diffracted to 2.5 Å, little phase information was obtained beyond 5 Å resolution. Solvent flattening using SOLOMON (Abrahams and Leslie, 1996) and DM (Cowtan and Main, 1996) was carried out in SHARP. Despite the poor quality of the initial phases, the map after solvent flattening was interpretable and could be used for model building (see Supplementary Figure S2). SHARP optimized the solvent content at 54.6%, with three copies in the asymmetric unit; these molecules were named chains 1, 2, and 3.

Initial model building was carried out in O (Jones et al., 1991), while COOT (Emsley and Cowtan, 2004) was employed for rebuilding. After several rounds of refinement using REFMAC5, it became clear in the F_O -F_C map that the asymmetric unit contained four copies instead of three. Large patches of "missing" density were

observed, indicating density as yet unaccounted for in the model. These patches suggested the presence of β-sheet structure. PHASER (McCoy et al., 2007) was then employed successfully to locate the fourth copy, called chain 4. Molecule 1 was used as the search model, and the positions of molecules 1, 2, and 3 were listed as "known" in the input file. Early rounds of refinement were undertaken using Hendrickson-Lattman coefficients obtained from SHARP, while late rounds employed no prior phase information. The structure was refined to final R_{cryst} and R_{free} values of 22.8% and 28.6% respectively with no residues in disallowed regions (Davis et al., 2007).

Table S1.

Crystal	Native	Mercury
Wavelength (Å)	0.9793	1.0
Resolution (Å)	77.9-2.2	115.5-2.5
R_{merge}[b]	0.056	0.109
R_{meas}[c]	0.061	0.119
<<I>/σ(<I>)>	20.9	13.4
Completeness (%)	99.4	99.0
Multiplicity	7.2	6.5
Wilson plot B (Å^2)	40.2	
**Phasing statistics**		
No. Sites/No expected	--	7/12
Rcullis anomalous/isomorphous	--	0.961/0.916
Phasing Power anomalous/isomorphous	--	0.413/0.579
<FOM> after SHARP	0.156	
<FOM> after SOLOMON	0.745	
**Refinement statistics**		
Resolution (Å)	77.9-2.2	
No. reflections/No. in R_{free}	33036/1746	
R_{cryst}/R_{free}[d]	0.228/0.286	
No. atoms protein/water	4399/171	
<B> protein (Å^2)	42	
Rmsd bond lengths (Å)	0.015	
Rmsd bond angles (°)	1.50	
**Ramachandran violations**[e]		
Most Favoured	96.2%	
Allowed	3.6%	
Disallowed	0.2%	
PDB ID	XXX	

For all supplementary crystallographic tables

[a] Values in parentheses apply to the high resolution shell
[b] $R_{merge} = \sum\sum_i |I_h - I_{hi}| / \sum\sum_i I_h$ where I_h is the mean intensity of reflection h.
[c] $R_{meas} = \sum\sqrt{(n/n-1)}\sum_i |I_h - I_{hi}| / \sum\sum_i I_h$, the multiplicity weighted R_{merge}
[d] $R = \sum(F_P - F_{calc}) / \sum F_P$
[e] From PROCHECK (Davis et al., 2007)

Supplemental Discussion

The survival of a Hrb deficient mouse, where the major observed abnormality was sterility (Kang-Decker et al., 2001), indicates that the trafficking of VAMP7 from the plasma membrane is not significantly altered as compared to the situation in wild type mice. This is likely due, in part at least, to the use of alternative pathways to bring about sufficient correct localisation of cargo as has been demonstrated for the lysosomal protein LAMP1 following depletion of AP-1,-3 and -4 (Janvier and Bonifacino, 2005). Deletion of some late endocytic SNAREs such as VAMP8 (Wang et al., 2007) and vti1b (Atlashkin et al., 2003) causes no major deleterious effect in whole mice, which points to a certain amount of redundancy between such SNAREs and this may further explain why disrupting VAMP7 endocytosis by deleting Hrb has little effect. A final factor in the survival of Hrb knock out mice is the presence of a highly similar gene HrbL, which is also present in homo sapiens and was unknown when (Kang-Decker et al., 2001) was published. Like Hrb, HrbL has an ArfGAP domain together with FXXFXXF, NPF and clathrin binding motifs. It also possesses an unstructured C-terminal tail containing a 20 residue stretch that is 76% conserved between Hrb and HrbL that in Hrb binds VAMP7, with all the key VAMP7 interacting residues conserved between the two proteins (see Supplementary Figure 9A). HrbL is able to localise to clathrin coated pits/vesicles as shown in Supplementary Figure 9B and the region of its C-terminal tail that is homologous to residues 136-176 in Hrb binds the VAMP7 longin domain with a K_D of around 100μM (data not shown). Clearly since HrbL is present and possibly upregulated in most cell types of the Hrb knockout mouse it could compensate for the lack of Hrb in VAMP7 endocytosis, so explaining its survival. The presence of HrbL in NRK cells as demonstrated by RTPCR at around a 30 fold lower than that of Hrb (data not

shown) may contribute to the relatively moderate increase in the amount of VAMP7-HA on the surface of NRK cells following Hrb depletion by siRNA (Figure 5A).

Figure S1. The sequences of Hrb

A) Protein sequence of human Hrb. The ARF-GAP domain is highlighted by a blue box, FXXFXXF AP2 appendage binding sequences are highlighted by a red box, predicted clathrin binding sequence is highlighted by a green box and VAMP7 binding sequence is highlighted by a gold box. EH-domain binding NPF motifs are indicated by asterisks. Accession number NM_004504

B) Clathrin binding. Rat brain detergent soluble lysate was incubated with equimolar amounts of either GST β2-adaptin appendage+hinge (positive control), GST Amphiphysin2 SH3 domain (negative control) and GST HrbHis$_6$(1-290) as in (Owen et al., 2000). GST fusion proteins were isolated using glutathione Sepharose and subjected to SDS-PAGE and transferred to nitrocellulose. The nitrocellulose was then blotted for clathrin using X22 monoclonal antibody (Abcam), monoclonal anti α-adaptin (BD Transduction labs) and antiHis$_6$ polyclonal antibody (Abcam) to show that the Hrb construct was intact to its C-terminus (data not shown). The blot showed that both GST β2-adaptin appendage+hinge and GST HrbHis$_6$(1-290) bound clathrin equally well wheras only GST HrbHis$_6$(1-290) bound AP2.

C) **Pull down of Hrb with the α-adaptin appendage domain.** HeLa cell detergent soluble lysate was incubated with either GST or GST fused to the α-adaptin C appendage domain. GST and GST-alpha-adaptin appendage domain proteins were isolated using glutathione Sepharose. Proteins bound to the Sepharose were subjected to SDS-PAGE and transferred to nitrocellulose. The nitrocellulose was then blotted for Hrb. Blot shows the amount of Hrb (1/20th of total lysate put into each pulldown) before pulldown (load), the amount of Hrb remaining in the lysate after pulldown (1/20th of total lysate) and Hrb isolated on the Sepharose beads after the pulldown.

D) The FXXFXXF sequences in Hrb function to bind α-adaptin. HeLa cell detergent soluble lysate was incubated with GST fused to various Hrb fragments each with a His_6 tag on the C-terminus. The His_6 tag (used in purification of the proteins) and mutations were added by PCR and the cDNAs subcloned into the SacII and XhoI sites of pGEX-3PLX. The HeLa cell lysate was incubated with the GST-fusion proteins and the GST-fusion proteins were isolated using glutathione Sepharose. Proteins bound to the Sepharose were subjected to SDS-PAGE and transferred to nitrocellulose. The nitrocellulose was then blotted for α-adaptin (monoclonal anti α-adaptin (clone8) BD Transduction Labs). Bottom blot shows $1/100^{th}$ of the amount of fusion protein put into each pulldown, by blotting for the C-terminal His_6 tag. Ctrl = 20 μg of cell lysate, acting as a positive control for the α-adaptin blotting. Note that the presence of one FXXFXXF binding seqeunce was sufficient for Hrb pull down α-adaptin, two sequences doubled the amount of α-adaptin bound and three motifs trebled the amount bound. Mutation of any FXXFXXF sequences quantitatively reduced binding by the predicted amount.

Figure S2. The $Hrb_{136-176}$/VAMP7 longin domain His6 chimera crystallised with four molecules in the asymmetric unit.

A Of the four molecules in the assymmetric unit (named A-D), the density for molecules B and D was the most continuous and unambiguous, allowing the best visualization of the interaction between the Hrb fragment and the longin domain.

The C and D molecules (blue and green) were self-contained: the Hrb fragment wrapped around the V7 longin domain molecule to which it was actually connected i.e an intramolecular interaction. However, in molecules A and B (purple and gold), the Hrb fragment extended across into a neighbouring V7 longin domain molecule.

B The density for the LysGly linker, which is part of Hrb, in molecule B was unambiguous and continuous, indicating clearly that Hrb stretched across to bind its neighbouring molecule, A.

C Representative solvent flattened experimental electron density maps of the Molecule A VAMP7 longin domain (purple) binding a portion of Hrb from Molecule B (gold).

D Interaction of GST Hrb(1-176) and GST control proteins with Wt and mutant versions of VAMP7 longin domain His_6 as indicated by western blotting of 'GST pull down' experiments with anti His_6 antibody. In agreement with ITC data (Figure 4) mutations in either protein reduce the levels of binding to those of controls.

E Interaction of GST VAMP7 longin domain and GST control proteins with Wt and mutant versions of Hrb(1-176)His_6 as indicated by western blotting of 'GST pull down' experiments with anti His antibody. In agreement with ITC data (Figure 4) mutations in either protein reduce the levels of binding to those of controls.

Figure S3. Expression of VAMP7-HA on the cell surface.

A) NRK cells stably expressing VAMP7-HA were transfected with water (Mock) or siRNA against Hrb (oligo1). Cells were fixed with paraformaldehyde. Cell-surface VAMP7 was visualised by labelling for the HA-epitope followed by an Alexa Fluor 488-conjugated secondary antibody. Increased VAMP7-HA on the surface following Hrb depletion can be seen as a marked increase in the intensity of HA staining on the cells' limiting membranes. Bar = 10 µm.

B) Ionomycin treatment of NRK cells results in cell surface expression of VAMP7-HA. NRK cells stably expressing full length human VAMP7-HA were fixed with paraformaldehyde and double labelled for the HA epitope and the lysosomal glycoprotein lgp120 (panels a and b). The HA tag did not alter the normal localization

of VAMP7 to lysosomes. Cells were also treated with (panels e and f) or without (panels c and d) 10 μM ionomycin for 10 min at 37 ˚C in HEPES buffered MEM before fixing with paraformaldehyde. The un-permeabilised cells were then stained for the HA epitope (panels c & e) or lgp120 (panels d & f). Increased VAMP7-HA on the surface following ionomycin treatment can be seen as a marked increase in the intensity of the HA staining on the cells' limiting membranes. Bar = 10 μm.

Figure S4 Depletion of clathrin by siRNA inhibits internalisation of VAMP7-HA

HeLa cells were transfected with water (Mock) or siRNA against clathrin heavy chain. Treatment with the siRNA against clathrin caused an approximately 70% reduction in clathrin levels as detected by western blotting **(A)** and by immunofluorescence (**B** panels a and e). In mock transfected cells there was little visible VAMP7-HA on the cell surface (panels b and c) whereas in cells treated with anti-clathrin siRNA, significant quantities of VAMP7-HA accumulated on the cell surface (panels f and g). Panels d and h show merges of a,b and c and of e,f, and g respectively.

Figure S5. Depletion of Hrb in HeLa cells by siRNA does not affect EGF uptake and degradation.

HeLa cells were transfected with water (Mock) or siRNA against Hrb, TSG101 and/or AMSH. Cells were incubated with ^{125}I-EGF and EGF uptake **(A)** and degradation **(B)** were measured as described in the Supplementary Materials and Methods section. n=5 for all conditions apart from AMSH (n=4) and TSG101 (n=1). Data are means ± SEM. The western blot shows the efficiency of Hrb depletion by siRNA (typical blot). Depletion of TSG101 and AMSH were used as controls because these respectively cause a reduction and increase in the rate of EGF degradation (Bowers et al., 2006).

Figure S6 Effect of disrupting the VAMP7 longin domain/Hrb interaction *in vivo*.

A) NRK cells stably transfected with ΔpMEP4 containing either wild type (WT) (a-f) or L43S/Y45S mutant (g-l) VAMP7-HA were treated with $CdCl_2$ to induce expression. They were then either fixed with paraformaldehyde (for surface staining of HA (a and g) or fixed and then permeabilised with Triton X-100 (for total staining of HA, d and j). After surface staining, cells were permeabilised if necessary and stained for the lysosomal membrane protein lgp110 followed by fluorescent labelled secondary antibodies. Cells stably expressing L43S/Y45S mutant VAMP7-HA have increased VAMP7-HA at the cell surface. The total amount of WT or L43S/Y45S mutant VAMP7-HA expressed in the transfected cells was the same (Supplementary Figure S6B), but the amount of HA tag was reduced when the WT construct was exposed to the proteolytic environment of lysosomes. c,f,i and l are merged and coloured versions of a and b, d and e, g and h, j and k respectively.

Images are confocal maximum intensity z-projections. Bars = 20 μm.

B) Similar expression levels of wild type VAMP7-HA and L43S/Y45S mutant VAMP7-HA in stably transfected NRK cells. Western blots of SDS-PAGE gel tracks containing equal protein loadings of stably transfected NRK cells with both anti-VAMP7 and anti-HA are shown. Whereas anti-VAMP7 staining was equal, anti-HA staining was reduced in the cells expressing WT VAMP7-HA because of increased lysosomal removal of the tag.

Figure S7. VAMP7 longin domain acts as an independent trfficking signal when fused to CD8.

NRK cells transiently expressing ΔpMEP4 constructs encoding CD8 (a, b, c) or chimaeras of CD8 followed by a flexible linker consisting of amino acids GSGGSG

connecting it to either the wild type (d,e,f) or L43S/Y45S mutant (g,h,i) longin domains of VAMP7. c,f and i are merged and coloured versions of a and b, d and e, g and h respectively. Lysosomes were observed using anti-Lgp110. The amount of CD8 on the surface and in internal structures was visualised by immunofluorescence using mouse anti-CD8

Images are confocal maximum intensity z-projections. Bars = 20 µm.

Figure S8. Schematic diagram of the role of Hrb as an endocytic adaptor for VAMP7.

Hrb simultaneously binds VAMP7 and AP2 appendage domains causing VAMP7 to be internalised as cargo by clathrin mediated endocytosis. Following delivery to the recycling endosome, VAMP7 can then be sorted by AP3 to its major intracellular location on late endocytic organelles. The SNARE complex in which VAMP7 was participating during its endocytosis must be disassembled and the other SNAREs to which it was bound recycled back to the cell's limiting membrane

Figure S9. HrbL co-localises with clathrin and Hrb in CCPs/CCVs. A) The sequences of Hrb and HrbL. Protein sequence alignment between human Hrb and Hrb-like protein (HrbL) sequences. The sequences are 41.6% identical. The ARF-GAP domain is highlighted by the dashedblue box. FXXFXXF motifs that bind to the alpha appendage of the adaptor protein-2 (AP-2) are boxed in red and the clathrin binding motifs are boxed in green. The VAMP7 binding sequences are boxed in gold. EH-domain binding NPF motifs are indicated by an asterisk. Identical residues between the two proteins are shaded black, conserved residues grey. The alignment was performed using ClustalW (http://www.ebi.ac.uk/clustalw/), using default parameters.

B) NRK cells were transiently transfected with a plasmid expressing EGFP-HrbL. Cells were pre-permeabilised with saponin then fixed with paraformaldehyde. Cells were labelled with mouse anti-clathrin (panel b) or goat anti-Hrb (panel f) followed by donkey anti goat or anti-mouse Alexa Fluor 555. The EGFP fluorescence in cells labelled for clathrin or Hrb is shown by panels a and e. Panels c and g are merged images and d and h are higher magnifications of the regions boxed in c and g. Images shown are confocal maximum intensity z-projections. HrbL can be seen to colocalise with both clathrin and Hrb. Bar = 10 μm.

A

```
MAASAKRKQEEKHLKMLRDMTGLPHNRKCFDCDQRGPTYVNMTVGSFVCTSCSGSLRGLN 60
PPHRVKSISMTTFTQQEIEFLQKHGNEVCKQIWLGLFDDRSSAIPDFRDPQKVKEFLQEK 120
YEKKRWYVPPEQAKVVASVHASISGSSASSTSSTPEVKPLKSLLGDSAPTLHLNKGTPSQ 180
SPVVGRSQGQQQEKKQFDLLSDLGSDIFAAPAPQSTATANFANFAHFNSHAAQNSANADF 240
ANFDAFGQSSGSSNFGGFPTASHSPFQPQTTGGSAASVNANFAHFDNFPKSSSADFGTFN 300
TSQSHQTASAVSKVSTNKAGLQTADKYAALANLDNIFSAGQGGDQGSGFGTTGKAPVGSV 360
VSVPSQSSASSDKYAALAELDSVFSSAATSSNAYTSTSNASSNVFGTVPVVASAQTQPAS 420
SSVPAPFGATPSTNPFVAAAGPSVASSTNPFQTNARGATAATFGTASMSMPTGFGTPAPY 480
SLPTSFSGSFQQPAFPAQAAFPQQTAFSQQPNGAGFAAFGQTKPVVTPFGQVAAAGVSSN 540
PFMTGAPTGQFPTGSSSTNPFL
```

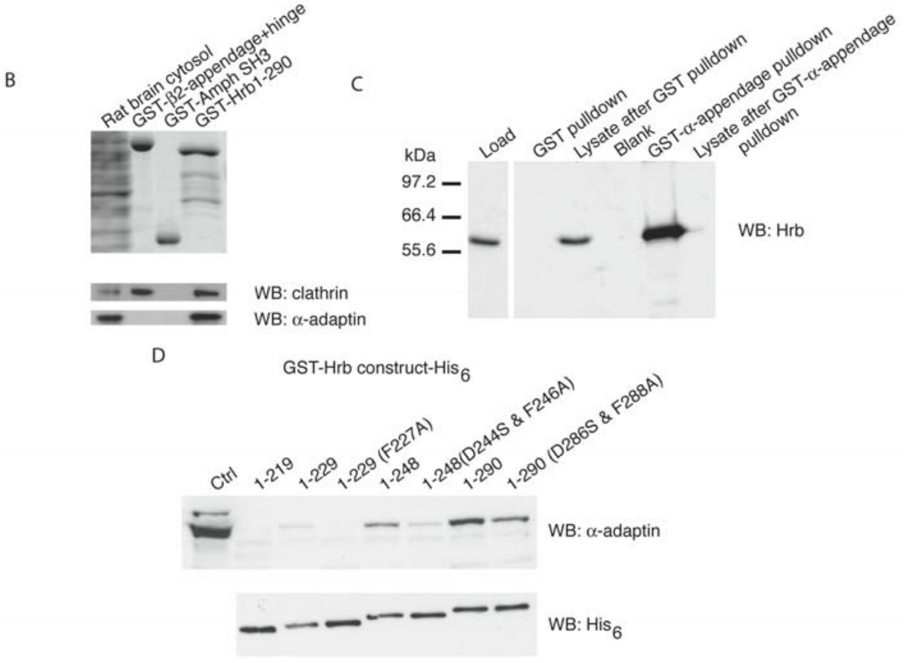

Pryor et al Supplementary Figure S1

A

B

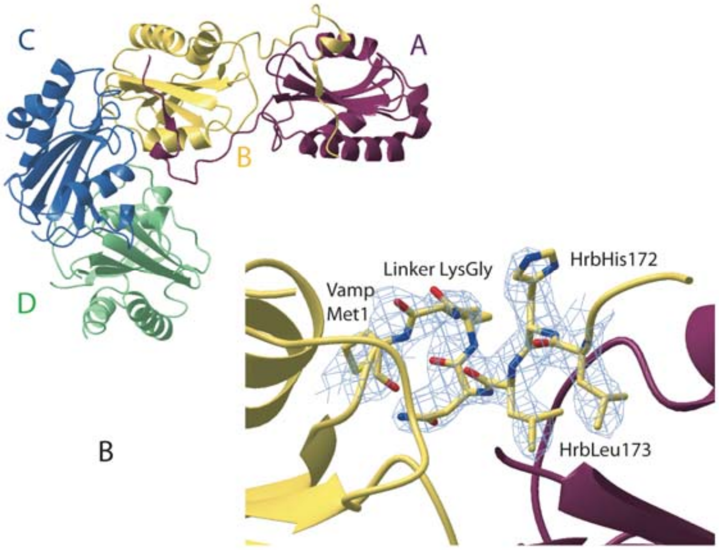

C

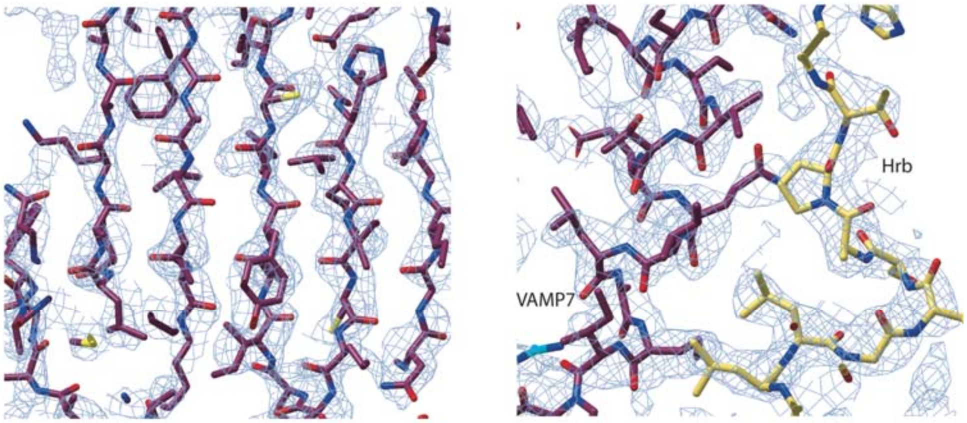

D

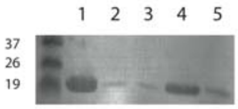

1 Wt VAMP7 LDHis$_6$
2 GST+Wt VAMP7 LDHis$_6$
3 GSTAmph2D+Wt VAMP7 LDHis$_6$
4 GSTHrb(1-176)+Wt VAMP7 LDHis$_6$
5 GSTHrb(1-176)+L43SY45SVAMP7 LDHis$_6$

E

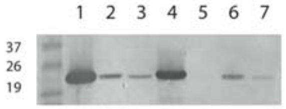

1 Wt Hrb(1-176)His$_6$
2 GST+Wt Hrb(1-176)His$_6$
3 GSTAmph2D+Wt Hrb(1-176)His$_6$
4 GSTVAMP7 LD+Wt Hrb(1-176)His$_6$
5 GSTVAMP7 LD+L160SL163SHrb(1-176)His$_6$
6 GSTVAMP7 LD+L163PL164PHrb(1-176)His$_6$
7 GSTVAMP7 LD+L171SL173SHrb(1-176)His$_6$

Pryor et al Supplementary Figure S2

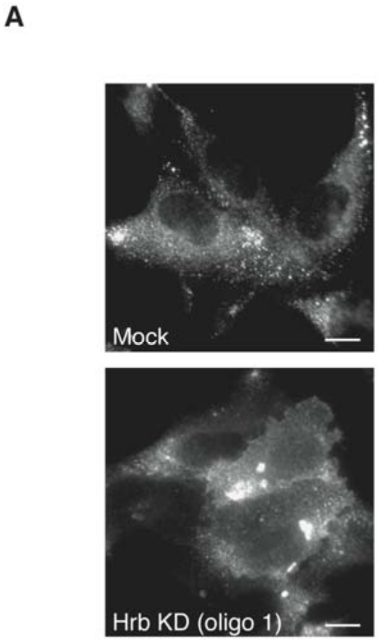

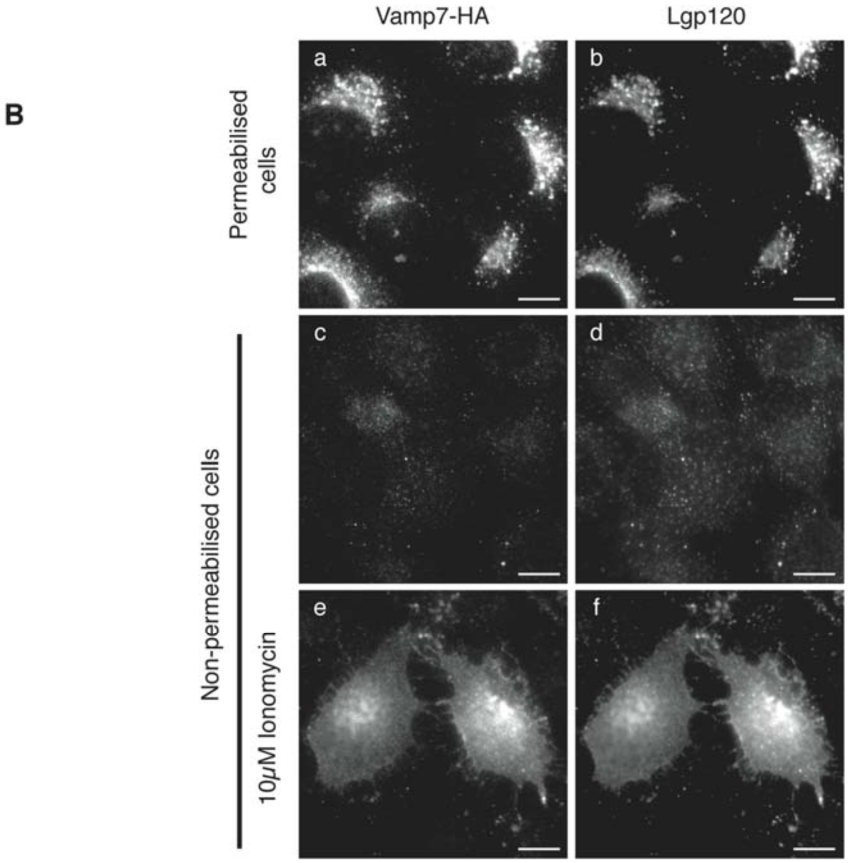

Pryor et al Supplementary Figure S3

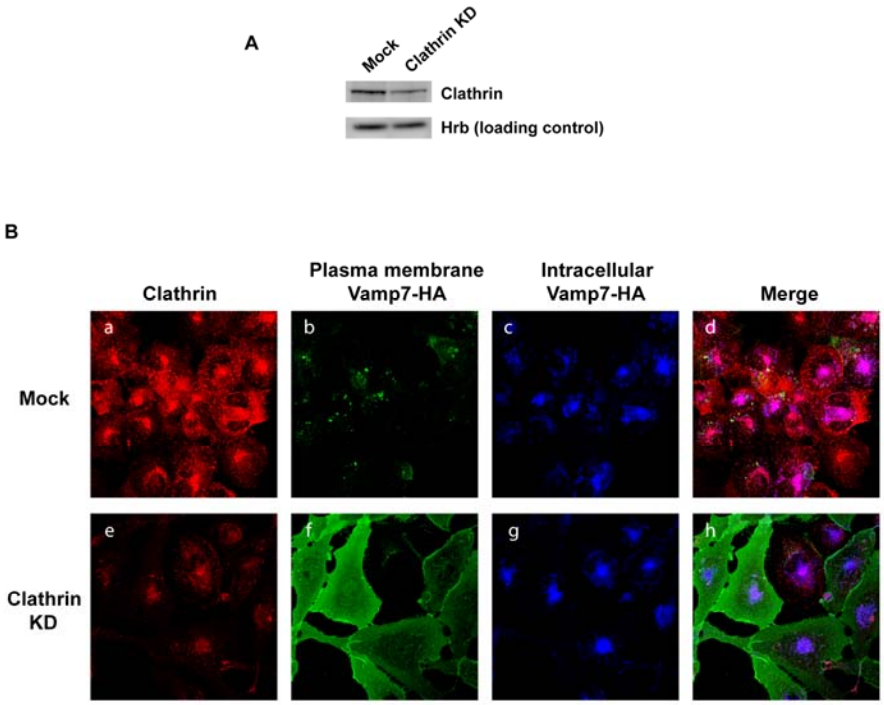

Supplementary figure S4

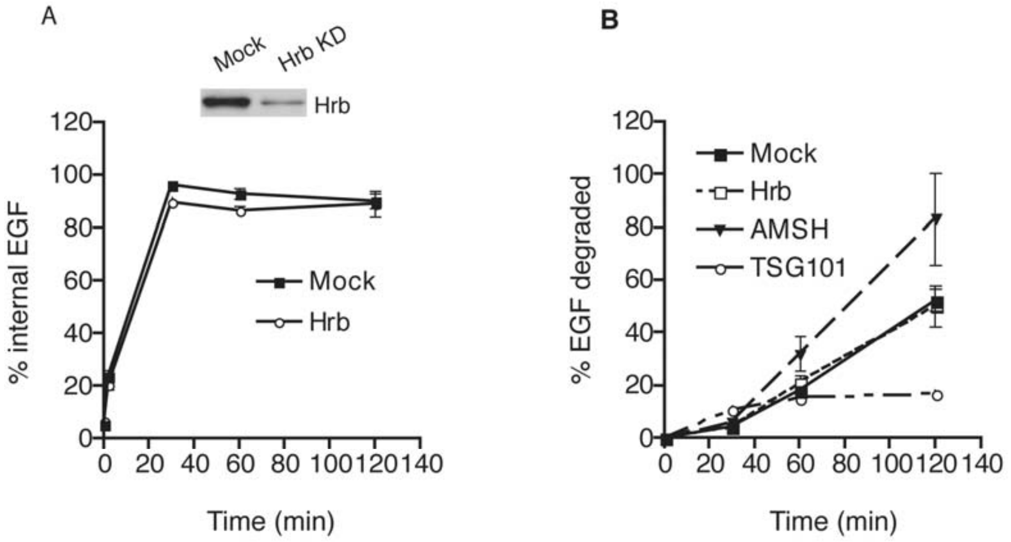

Pryor et al Supplementary Figure S5

A

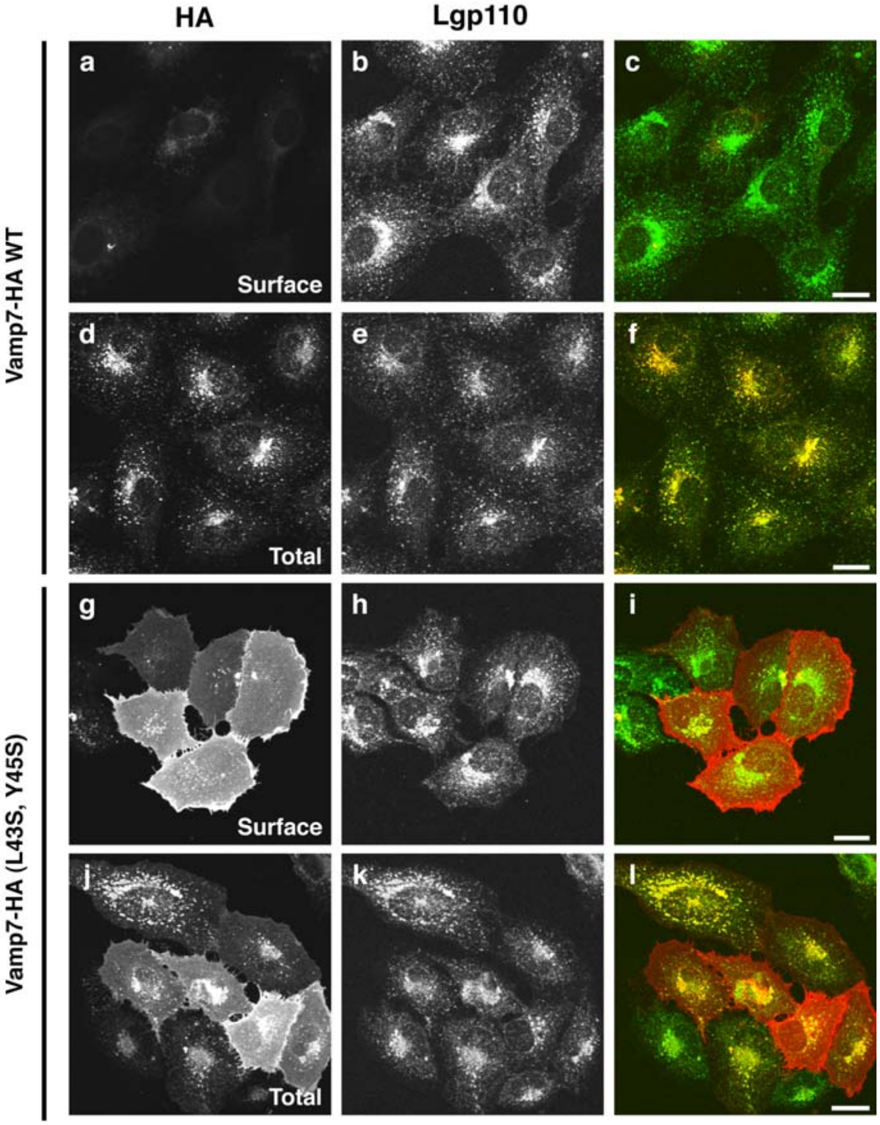

B

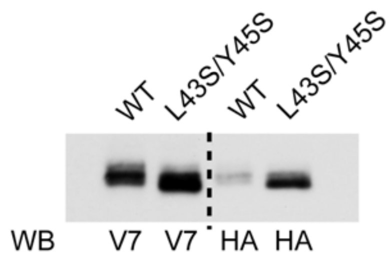

Pryor et al Supplementary figure S6

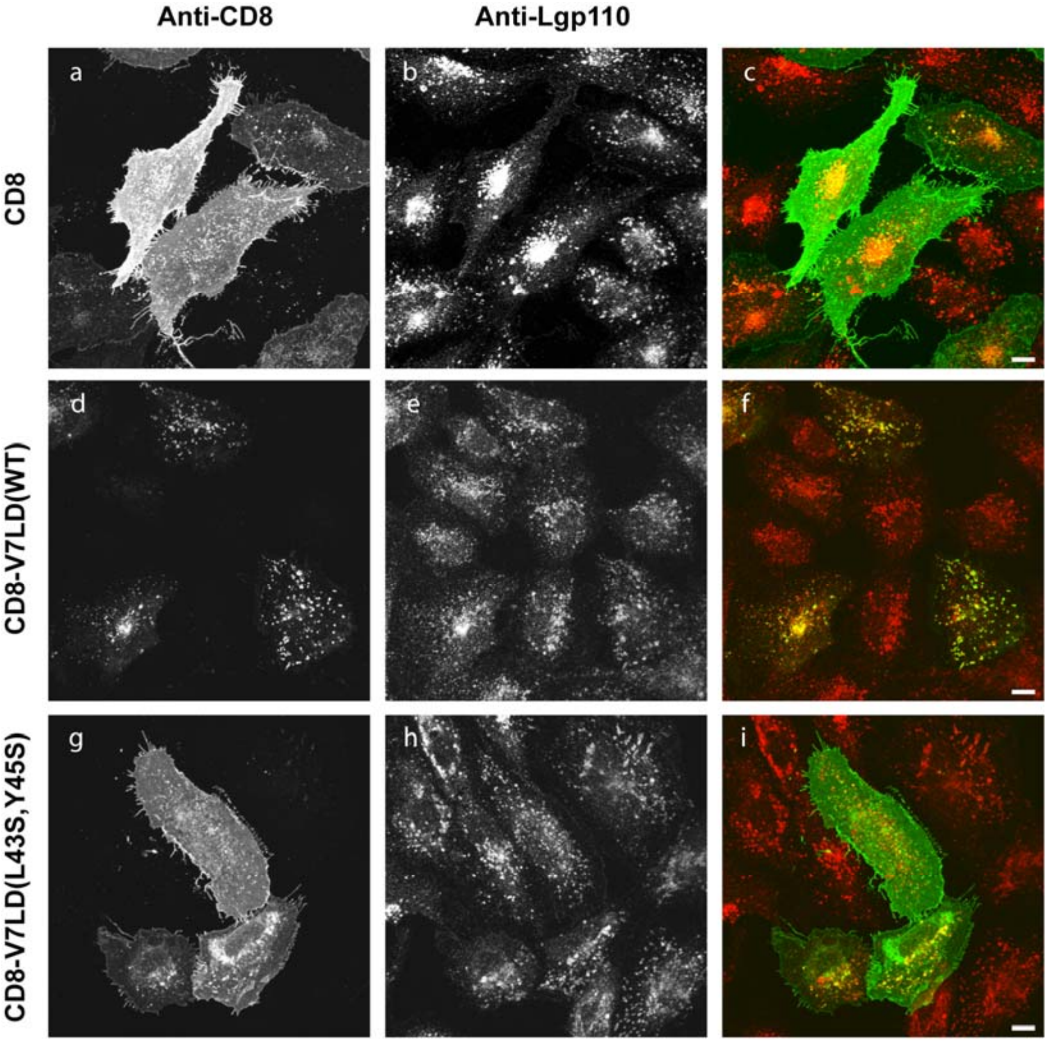

Pryor et al Supplementary figure S7

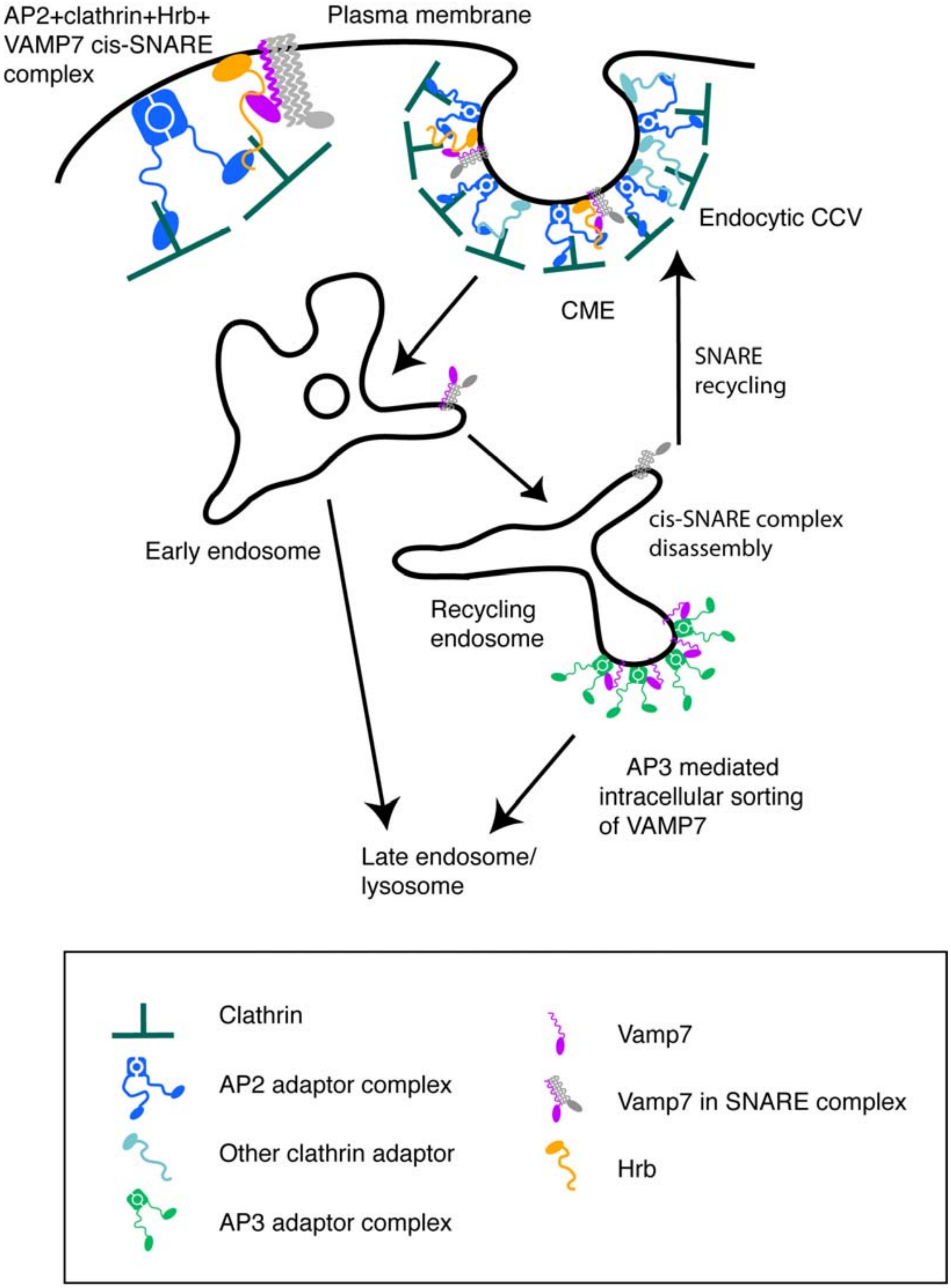

Pryor et al Figure S8

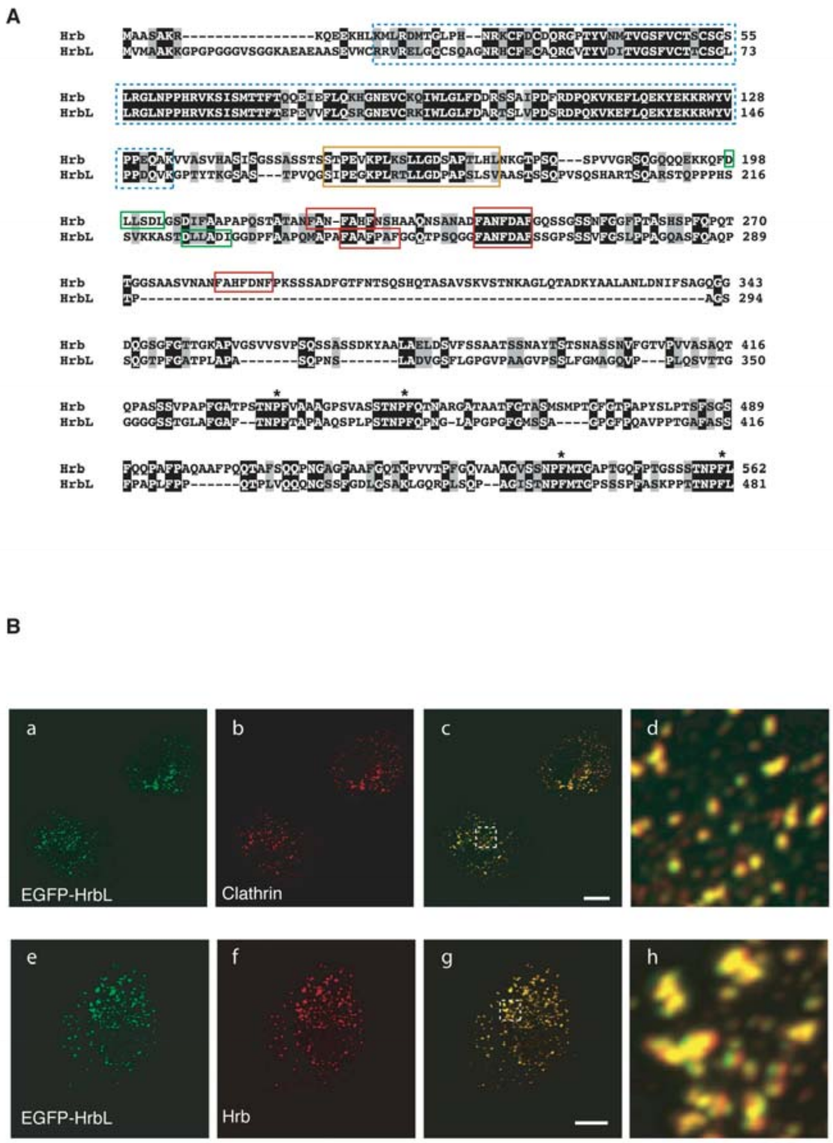
A
B
a
b
c
d
e
f
g
h
EGFP-HrbL
Clathrin
EGFP-HrbL
Hrb
